# A Large Detection-Range Plasmonic Sensor Based on An H-Shaped Photonic Crystal Fiber

**DOI:** 10.3390/s20041009

**Published:** 2020-02-13

**Authors:** Haixia Han, Donglian Hou, Lei Zhao, Nannan Luan, Li Song, Zhaohong Liu, Yudong Lian, Jianfei Liu, Yongsheng Hu

**Affiliations:** 1 Tianjin Key Laboratory of Electronic Materials and Devices, School of Electronics and Information Engineering, Hebei University of Technology, Tianjin 300401, China; 201821902029@stu.hebut.edu.cn (H.H.); 201921902013@stu.hebut.edu.cn (D.H.); 201721902001@stu.hebut.edu.cn (L.Z.); songli@hebut.edu.cn (L.S.); lzh@hebut.edu.cn (Z.L.); ydlian@hebut.edu.cn (Y.L.); jfliu@hebut.edu.cn (J.L.); 2 State Key Laboratory of Luminescence and Applications, Changchun Institute of Optics, Fine Mechanics and Physics, Chinese Academy of Sciences, Changchun 130033, China; huyongsheng@ciomp.ac.cn

**Keywords:** fiber optics sensors, photonic crystal fiber, refractive index sensor, surface plasmon resonance

## Abstract

An H-shaped photonic crystal fiber (PCF)-based surface plasmon resonance (SPR) sensor is proposed for detecting large refractive index (RI) range which can either be higher or lower than the RI of the fiber material used. The grooves of the H-shaped PCF as the sensing channels are coated with gold film and then brought into direct contact with the analyte, which not only reduces the complexity of the fabrication but also provides reusable capacity compared with other designs. The sensing performance of the proposed sensor is investigated by using the finite element method. Numerical results show that the sensor can work normally in the large analyte RI (*n*_a_) range from 1.33 to 1.49, and reach the maximum sensitivity of 25,900 nm/RIU (RI units) at the *n*_a_ range 1.47–1.48. Moreover, the sensor shows good stability in the tolerances of ±10% of the gold-film thickness.

## 1. Introduction

The surface plasmon resonance (SPR) phenomenon has been widely studied and applied in medical diagnostics, environmental monitoring, and biochemical research due to its high sensitivity, real-time and label-free monitoring [1,2,3,4,5,6]. Most commercial SPR sensors are based on a prism structure. However, because these structures require optical and mechanical components, they have the disadvantages of bulky configuration and high cost, which limits their practical application [1,2,3,4]. Using optical fiber-based SPR instead of a bulky prism can improve the integration degree. In these fiber-based designs, the fiber jacket is physically or chemically removed to expose the core to the sensing region in order to enhance the coupling between the core mode and the surface plasmon polariton (SPP) modes [1,2,3,4,5,6]. The laborious process will make the sensor more fragile. In recent years, many researchers have focused on different types of photonic crystal fiber (PCF)-based SPR sensor [7,8,9,10,11,12,13,14,15,16,17,18,19,20,21,22,23,24]. By introducing air holes in the core area of the PCF, the effective refractive index (*n*_eff_) of the core mode can be reduced, thus easily realizing the phase matching between the core mode and the SPP modes [14,15,16,17,18]. These PCF-based SPR sensors have the advantages of miniaturization, high sensitivity and multi-parameter measurement, which make them more competitive in SPR sensing applications [7,8,9,10,11,12,13,14,15,16,17,18,19,20,21,22,23,24]. However, currently, PCF-based SPR sensors have two principal challenges: the first problem is the difficult process of the sensor’s fabrication, such as metal coating and analyte filling. In practice, the holes in these sensors are very small in size, generally in the order of micrometers [8,11,13,14,15,16,17,20,21,24]. Therefore, it is difficult to coat them with metal film uniformly and to fill them with liquid analyte within the predetermined parameters. Furthermore, these processing methods make it difficult to reuse for the replacement of analyte; Another problem is the narrow refractive index (RI) range of the sensor detection, either employing low RI or high RI PCF-SPR sensors [14,15,16,17,18,19,20,21,22,23]. In these sensors, in order to meet the total reflection condition, the highest RI value detected by the low RI PCF-SPR sensors is lower than that of the fiber materials [14,15,16,17,18,19], whereas the lowest RI value detected by the high RI PCF-SPR sensors must be higher than that of the fiber materials [20,21,22,23].

**Figure 1:**
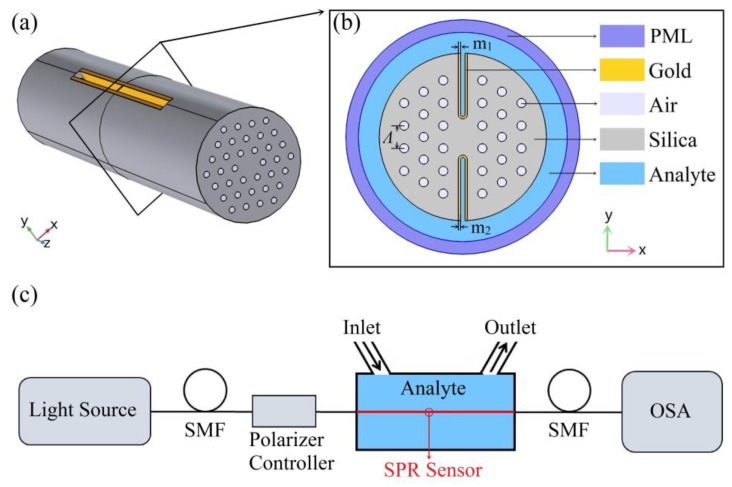
(**a**) Schematic diagram of the proposed H-shaped photonic crystal fiber-based surface plasmon resonance (PCF-SPR) sensor; (**b**) cross-section of the SPR sensor; (**c**) experimental setup of the SPR sensor for refractive index (RI) detection.

In this paper, we propose an open structure PCF-SPR sensor design that not only solves the problem of sensor fabrication but also can detect the RI over a larger range, either higher or lower than that of the fiber materials. The basic geometry of the PCF follows the H-shaped structure as shown in Figure 1. This open structure avoids coating in the holes with metal film and can be in direct contact with the analyte, thus reducing the manufacturing complexity and offering reusable capability. We use the finite element method based commercial COMSOL software to analysis the electromagnetic modes and the sensing performance of the sensor.

## 2. Structure Design and Principle

The schematic diagram of the proposed H-shaped PCF-SPR sensor is shown in Figure 1. The three layers of air holes are arranged in a hexagonal geometry, forming an H-shaped structure with symmetrical grooves. The gold film as a plasmonic material is placed on the internal surface of the grooves. These grooves are particularly advantageous for metal coating, and have the characteristics of good accessibility and easy replacement of analyte. The special structure can be made by femtosecond laser micromachining [25], focused ion-beam milling [26,27], or chemical etching of the original side-hole PCF [28,29].

In our simulation, the distance between the holes is Λ = 8 μm and the diameters of the cladding holes is 0.5Λ, respectively. The opening depth is set to *h* = 3Λ, the widths of the two grooves are both 0.5Λ, and the thickness of gold film is *m*_1_ = *m*_2_ = 40 nm. The RI of air is 1, and the RI of the fiber materials is fixed at 1.45 in order to clearly show that the RI of the analyte can either be higher or lower than that of the fiber materials. In addition, the complex dielectric constant (Ɛ(ω)) of gold is defined by using the Drude–Lorentz model [30]:

(1) $$\varepsilon \left(\omega \right)=\varepsilon_{\infty }-\frac{\omega_{D}^{2}}{\omega \left(\omega +j\gamma_{D}\right)}+\frac{\Delta \varepsilon \cdot \Omega_{L}^{2}}{\left(\omega^{2}-\Omega_{L}^{2}\right)+j\Gamma_{L}\omega }$$

where *ω*_D_ represents the plasma frequency, *ϒ*_D_ is the damping frequency. Ω_L_, Γ_L_ and Δ*ε* can be interpreted as the oscillator strength, spectral width of the Lorentz oscillators, and weighting factor [30]. In addition, we use the artificial boundary condition of the outermost perfect matching layer (PML) to absorb the radiant energy as shown in Figure 1b [13,19,31].

**Figure 2:**
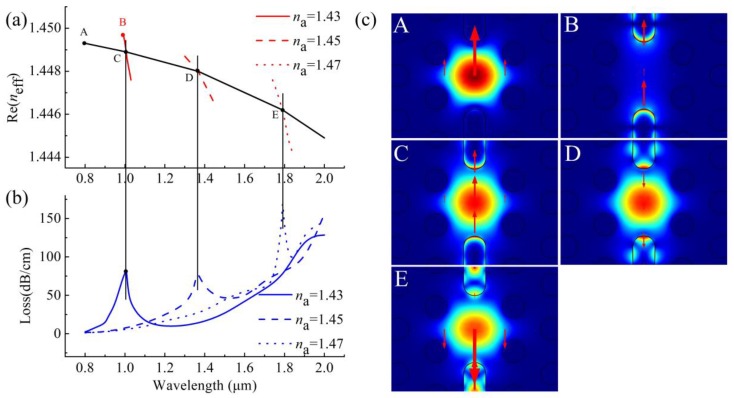
(**a**) Re (*n*_eff_) curves of the *y*-polarized core mode (black solid curve) and *y*-polarized surface plasmon polariton (SPP) mode at *n*_a_ = 1.43, 1.45, and 1.47 (red solid, red dashed and red dotted curves); (**b**) loss spectra of the *y*-polarized core mode at *n*_a_ = 1.43, 1.45, and 1.47; (**c**) *y*-polarized electric field distributions of core mode and SPP mode at specific wavelengths (A for core mode at 800 nm with *n*_a_ = 1.43, B for SPP mode at 992 nm with *n*_a_ = 1.43, C for core mode at 1006 nm with *n*_a_ = 1.43, D for core mode at 1367 nm with *n*_a_ = 1.45 and E for core mode at 1791 nm with *n*_a_ = 1.47), arrows indicate polarized direction of electric fields.

Because the core mode with the electric field predominantly orthogonal to the metal surface can be more readily coupled to the SPP modes on the metal surface [8,9,18,22,32,33,34], in this design the *y*-polarized core mode has a better SPR phenomenon than the *x*-polarized core mode, thus providing a better sensing performance. In what follows, we mainly investigate the sensing performance of the *y*-polarized core mode. In Figure 2a, we plot the real part of the *n*_eff_ (Re(*n*_eff_)) curves of the *y*-polarized core mode and SPP modes when the RI of the analyte (*n*_a_) is 1.43, 1.45, and 1.47, respectively, to indicate that the proposed sensor has potential for RI sensing that can be higher or lower than the RI of fiber materials. The black solid line represents the Re(*n*_eff_) of *y*-polarized core mode, whereas the red solid, red dashed and red dotted lines respectively represent the Re(*n*_eff_) of *y*-polarized SPP modes at *n*_a_ = 1.43, 1.45, and 1.47, as shown in Figure 2a. Take the case of *n*_a_ at 1.43; the core mode and SPP mode are coupled where their *n*_eff_ curves intersect (C point in Figure 2a). Losses of the core mode increase sharply near this intersection (resonance wavelength) as shown in Figure 2b, because the energy of the core mode transfers to SPP modes which can be observed from the inset C in Figure 2c. Similar processes of this energy transfer also occur at the intersections D and E when the *n*_a_ is 1.45 and 1.47, respectively, which can be seen from Figure 2b and the insets D and E in Figure 2c. In Figure 2a,b, we also observed that the wavelength of resonance peak is 1006 nm, 1367 nm, and 1791 nm when *n*_a_ is 1.43, 1.45, and 1.47, respectively. That is, as *n*_a_ increases, the resonance peak shifts to longer wavelengths. This peak behavior can be used to measure the change of the analyte RI.

## 3. Results and Discussion

### 3.1. Sensing Performance

**Figure 3:**
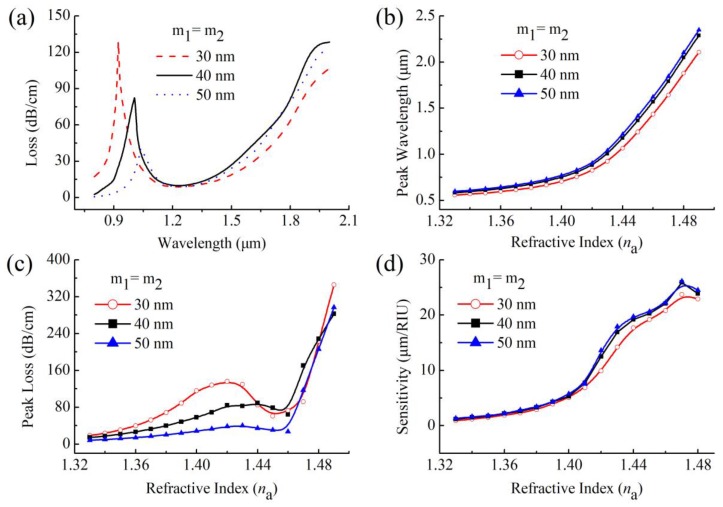
(**a**) Loss spectra of the SPR sensor at *n*_a_ = 1.43 when the thickness of gold film (*m*_1_ = *m*_2_) is 30 nm,40 nm, and 50 nm; (**b**) peak wavelengths, (**c**) peak loss and (**d**) sensitivities of the SPR sensor at various *n*_a_ when the thickness of gold film (*m*_1_ = *m*_2_) is 30 nm, 40 nm, and 50 nm.

By measuring the shift of the peak wavelength, the change in *n*_a_ can be determined. The sensitivity of the sensor is given by [8,9,12,14,15,16,17,18,19,20,21,22,24]:

(2) $$S_{\lambda }=\left(nm/RIU\right)=\frac{\Delta \lambda_{peak}}{\Delta n_{a}}$$

where, Δ*λ_peak_* denote the shift of peak wavelength and Δ*n*_a_ is the change of *n*_a_. To give an example in Figure 2b, *n*_a_ changes from 1.45 to 1.47, and the corresponding peak wavelength shifts Δ*λ_peak_* = 424 nm, which means that the sensitivity of the sensor is 21,200 nm/RIU (RI units). Figure 3b,d respectively depict the peak wavelengths and sensitivities curves of the sensor when the *n*_a_ changes from 1.33 to 1.49. On the whole, as *n*_a_ increases with the same Δ*n*_a_, the Δ*λ_peak_* of the peak wavelength also becomes larger, according to Equation (2), the sensitivity of the sensor also increases correspondingly, reaching a maximum value of 25,900 nm/RIU when the *n*_a_ range of 1.47–1.48 as shown in Figure 3d. This sensing performance make it very suitable to measure some high RI organic chemical analytes, such as chloroform toluene or benzene [35].

### 3.2. Gold-Film Thickness

The thickness of the metal film is a significant parameter affecting the resonance coupling between the core mode and the SPP modes [14,15,16]. Figure 3a shows the loss spectra of the *y*-polarized core mode at *n*_a_ = 1.43 for various thicknesses of gold film (*m*_1_ = *m*_2_) 30 nm, 40 nm, and 50 nm. As can be seen from the figure, with the thickness of the gold film becoming thicker, the corresponding peak wavelength shifts towards longer wavelengths and, meanwhile, the peak loss shows a downward trend. This peak behavior is also consistent with that at the other *n*_a_, as shown in Figure 3b,c. The main reason for these phenomena is that the increase of gold-film thickness increases the distance between the core area and the gold surface, and only at longer wavelengths can the electric field of the core mode penetrate the gold film, coupling with the SPP modes on the gold surface. The increase of gold-film thickness also reduces the coupling intensity between the core mode and the SPP modes, thus reducing the peak loss. Note that the curves of peak loss appear to significantly decline in the vicinity of 1.45. This is due to the fact that the *n*_a_ is not very different from the RI of the background material, the confinement of the core mode becomes weaker and more energy of the core mode leaks out to the analyte region. To further study the effect of gold-film thickness on sensing performance, we also present the sensitivities of the sensor at different *n*_a_ with gold-film thickness at 30 nm, 40 nm, and 50 nm in Figure 3d. In general, the sensor shows a higher sensitivity with the thicker gold film, especially at the larger *n*_a_ range.

### 3.3. Fabrication Tolerance

**Figure 4:**
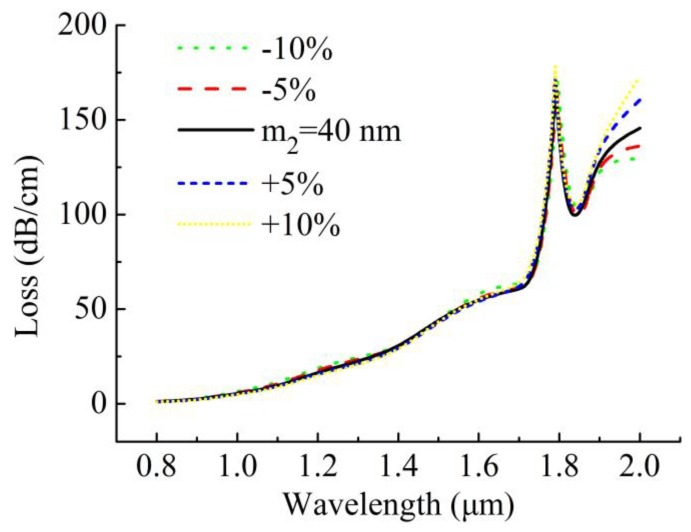
Loss spectra of *y*-polarized core mode at *n*_a_ = 1.47 when *m*_1_ is fixed at 40 nm and *m*_2_ changes in the range of ±10%.

Coating with gold film on surfaces of such grooves in this design is much simpler than that on inner surfaces of the air holes in the other designs [13,14,15,16,17,20,24]. In actual manufacturing, however, it is difficult to accurately deposit the gold film on the surface of the two grooves under the same thickness. It is necessary to investigate the effect of the fabrication tolerances of the gold film on the sensing performance of the sensor. Due to the symmetry of its structure, we only consider the case in which the variation of ±10% of *m*_2_ affects the sensing performance of the proposed sensor when the *m*_1_ is fixed to 40 nm. Figure 4 shows the loss spectra with slight variation of ±10% of *m*_2_ at *n*_a_ = 1.47. When *m*_2_ varies from -10% to +10%, the loss spectra present a slight change.

In order to further investigate the effect of the *m*_2_ on sensing performance of the proposed sensor, we summarize the peak wavelengths, peak losses and sensitivities for variation of ±10% of *m*_2_ in Table 1 when *m*_1_ is fixed at 40 nm and *n*_a_ changes from 1.33 to 1.49. In general, as *m*_2_ changes from −10% to +10%, the peak wavelength shows a slight decrease in the low *n*_a_ range and then follows an opposite trend in the high *n*_a_ range, and the peak loss shows a small decrease in the whole *n*_a_ range. Those slight changes have negligible influence on the sensitivities of the sensor as shown in Table 1, which indicates that the sensor has good stability within the variation of ±10% fabrication tolerances for the thickness of the gold film and low requirements of manufacturing precision.

## 4. Conclusions

In this paper, we propose a novel design of an open structure H-shaped PCF with a capability of a wide RI-detection range. The gold film and the analyte are placed on the surface of the grooves in the H-shaped PCF, making the fabrication of the sensor much easier than traditional PCF-SPR sensors in which the gold film and the analyte are placed in the air holes. The results demonstrate that the proposed sensor can work well in a large *n*_a_ range, and has good stability within tolerances of ±10% of the gold-film thickness. Compared with the other types of SPR sensor, our proposed SPR sensor has the advantages of convenient operation, simple fabrication, good stability, large detection range, high sensitivity, and is reusable, which make it more competitive in physical-, biological- and chemical-sensing fields.

## Figures and Tables

**Table 1:** Summary of peak wavelengths, peak losses and sensitivities of *y*-polarized core mode at the *n*_a_ range of 1.33–1.49, when *m*_1_ is fixed at 40 nm and *m*_2_ changes in the range of ±10%.

		Peak Wavelength(μm)					Peak Loss (dB/cm)					Sensitivities (μm/RIU)				
	*m*_2_	−10%	−5%	40nm	5%	10%	−10%	−5%	40nm	5%	10%	−10%	−5%	40nm	5%	10%
*n_a_*																
1.33		0.578	0.581	0.583	0.585	0.586	14.851	14.819	14.337	13.565	12.675	1.4	1.2	1.2	1.2	1.2
1.34		0.592	0.593	0.595	0.597	0.598	18.086	18.128	17.519	16.495	15.298	1.2	1.4	1.5	1.5	1.5
1.35		0.604	0.607	0.610	0.612	0.613	22.082	22.278	21.509	20.126	18.534	1.6	1.8	1.7	1.7	1.7
1.36		0.62	0.625	0.627	0.629	0.630	27.026	27.416	26.461	26.298	22.492	2.1	2.1	2.2	2.1	2.2
1.37		0.641	0.646	0.649	0.65	0.652	33.132	33.740	32.525	32.204	27.305	2.5	2.6	2.6	2.7	2.6
1.38		0.666	0.672	0.675	0.677	0.678	40.562	41.374	39.819	36.646	33.059	3.3	3.3	3.3	3.4	3.4
1.39		0.699	0.705	0.708	0.711	0.712	49.647	50.440	48.404	44.363	44.066	4.1	4.2	4.3	4.2	4.2
1.40		0.740	0.747	0.751	0.753	0.754	60.516	61.043	58.133	53.303	48.008	5.6	5.4	5.3	5.6	5.6
1.41		0.796	0.801	0.804	0.809	0.810	72.820	72.826	68.803	63.328	57.383	7.9	7.3	7.7	7.4	7.3
1.42		0.875	0.874	0.881	0.883	0.883	88.037	83.011	84.088	73.576	68.881	10.4	10.6	12.5	12.3	12.4
1.43		0.979	0.980	1.006	1.006	1.007	159.55	90.628	82.598	86.326	86.312	15.1	16.8	16.9	17.1	17.0
1.44		1.130	1.148	1.175	1.177	1.177	133.42	152.06	89.12	141.24	110.87	20.3	20.9	19.2	19.8	19.8
1.45		1.333	1.357	1.367	1.375	1.375	61.504	75.280	78.673	84.268	55.715	23.8	21.4	20.3	19.4	19.4
1.46		1.571	1.571	1.570	1.569	1.569	60.016	68.644	63.983	44.704	40.176	22.2	22.2	22.1	22.0	22.1
1.47		1.793	1.793	1.791	1.789	1.790	171.65	167.58	170.16	173.26	179.64	26.2	26.0	25.9	25.9	25.8
1.48		2.055	2.053	2.050	2.048	2.048	242.29	253.29	263.00	254.08	244.21	23.8	23.8	23.9	23.8	23.7
1.49		2.293	2.291	2.289	2.286	2.285	304.38	302.30	282.80	299.62	283.39					
